# 584 Improving the Quality of Trainee Burn Size Estimation on Admission to a Regional Burn Center

**DOI:** 10.1093/jbcr/irae036.218

**Published:** 2024-04-17

**Authors:** Jack Bullis, Meghna Kurup, Lori Mickelson, Angela Gibson, Lee D Faucher, Lauren B Nosanov

**Affiliations:** University of Wisconsin, Madison, WI; University of Wisconsin (UW Health), Madison, WI; University of Wisconsin School of Medicine and Public Health, Madison, WI; University of Wisconsin, Middleton, WI; University of Wisconsin, Madison, WI; University of Wisconsin (UW Health), Madison, WI; University of Wisconsin School of Medicine and Public Health, Madison, WI; University of Wisconsin, Middleton, WI; University of Wisconsin, Madison, WI; University of Wisconsin (UW Health), Madison, WI; University of Wisconsin School of Medicine and Public Health, Madison, WI; University of Wisconsin, Middleton, WI; University of Wisconsin, Madison, WI; University of Wisconsin (UW Health), Madison, WI; University of Wisconsin School of Medicine and Public Health, Madison, WI; University of Wisconsin, Middleton, WI; University of Wisconsin, Madison, WI; University of Wisconsin (UW Health), Madison, WI; University of Wisconsin School of Medicine and Public Health, Madison, WI; University of Wisconsin, Middleton, WI; University of Wisconsin, Madison, WI; University of Wisconsin (UW Health), Madison, WI; University of Wisconsin School of Medicine and Public Health, Madison, WI; University of Wisconsin, Middleton, WI

## Abstract

**Introduction:**

Accurate estimation of total body surface area (TBSA) with documentation on a Lund Browder (LB) chart is critical in guiding burn treatment and determining proper level of care. Recent quality improvement (QI) review of a series of patients admitted to our regional burn center has raised concerns about inaccurate admission TBSA estimations. The purpose of this study is to evaluate initial LB charts and subsequent iterations throughout the hospitalization in order to identify opportunities to improve TBSA fidelity and subsequent delivered care.

**Methods:**

The Burn Center institutional registry was queried for all patients admitted over a three-month period. After exclusion of those with non-burn etiologies, retrospective chart review was performed for remaining patients to gather data on demographics, injury characteristics, and clinical outcomes. TBSA estimations assessed included outside hospital, Emergency Department, burn center admission, and all other completed LB diagrams from the index hospitalization. Patients were subcategorized by time of admission and whether admitting attending staff was primarily specialized in Burns. Percentage difference between admission and final LB was computed to analyze under- and over-estimation tendencies.

**Results:**

After exclusions, 100 patients – 53 adult and 47 pediatric cases – admitted 5/2023-8/2023 were included for analysis. The patient cohort was young (median 19 years) and predominantly male (65.0%). Most common burn etiologies were flame (33.0%), scald (28.0%), and contact (24.0%). Night/weekend admissions by the covering Acute Care surgeons comprised 54.0%. Admission total TBSA was equivalent to final TBSA for 75.0%, while 14.0% initially underestimated and 11.0% overestimated. Among patients with TBSA ≧ 20.0%, 44.4% had consistent initial to final TBSA while 55.6% underestimated, all of whom were admitted by a non-Burn physician at night or on the weekend. In this large burn cohort, the percent difference of underestimation ranged from 4.1-21.9%.

**Conclusions:**

Lack of experience and familiarity with burn size estimation may make LB completion challenging, with associated clinical consequences. In our patient population this was particularly a problem in larger burns admitted by covering Acute Care Surgery staff. While over-estimation by referring facility and Emergency Medicine providers is common, admission initial LBs tended to underestimate in these cases. Further QI work is planned to improve the accuracy of initial TBSA calculations through use of a physician-nursing huddle protocol.

**Applicability of Research to Practice:**

Inaccurate estimations of TBSA, especially upon admission, can lead to life-threatening consequences from inappropriate resuscitation and mis-triage. Our goal is to target initial LB completion processes to improve care while also fostering interprofessional teamwork.

